# Inverted human umbilical artery as a 3D scaffold for sciatic nerve regeneration in rats

**DOI:** 10.1007/s10561-022-10006-8

**Published:** 2022-05-03

**Authors:** Flore-Anne Lecoq, Laurence Barnouin, Ludovic Ardouin, Daniel Hartmann, Laurent Obert

**Affiliations:** 1 Institut de la Main Nantes Atlantique, Elsan Santé Atlantique, Saint Herblain, France; 2 Tissue Bank of France (TBF), 6 rue d’Italie, 69780 Mions, France; 3 Novotec, 11 rue Edison, 69500 Bron, France; 4 CHRU Besançon, Besançon, France

**Keywords:** Posttraumatic peripheral nerve repair, Microsurgery, Nerve conduit, Umbilical artery

## Abstract

Treatment of peripheral nerve injuries (PNIs) remains a challenge. Interposing a graft delivers better regenerative outcomes. Autografts present major drawbacks which have given rise to the development of alternatives such as artificial scaffolds, some of which are very promising. This study was designed to investigate the potential use of an inverted human umbilical cord artery (iHUA) as a 3D scaffold nerve chamber, for nerve regeneration after transection of the sciatic nerve (SN) in rats. Rats underwent surgical SN transection in their right hindlimb, followed by suture of the device at the resected stumps. Local tolerance, insert biodegradability and nerve reconstruction over time were thoroughly studied by histopathological and morphometric analysis, completed by functional test assessment of sensitivity and motricity recovery. We have demonstrated that nerve reconstruction in the presence of an iHUA insert is effective. The device is well tolerated and highly biodegraded. Although the regenerated nerve is still immature at the end of our study, signs of sensitivity and partial functional recovery were witnessed, confirming our histological findings. Our results support the potential clinical use of iHUA as a 3D scaffold to bridge nerve discontinuity and guide axonal regrowth in selected cases of PNIs.

## Introduction

Peripheral nerve injury (PNI) can result in severe sensory and motor dysfunction and remains a medical challenge. Although the peripheral nervous system has an intrinsic regeneration capability, function is rarely restored spontaneously. Great efforts have been made to rebuilt and to restore motor and sensory functions, but still proper healing remains challenging. Strategies adopted for surgical repair of PNIs depend largely on the size and the type of the nerve lesion (Tezcan 2017; Alvites et al. 2018; Caillaud et al. 2019). Direct tension-free neurorrhaphy is the current “gold standard” for reconstruction after complete nerve transection without significant damage, especially for injuries less than 5 mm long, while larger wounds are generally treated with autologous nerve graft approach (Siemionow and Brzezicki 2009; Xie et al. 2014; Tezcan 2007). It is well established that interposing a graft between proximal and distal nerve stumps deliver better regenerative outcomes, as it offers abundant cellular support for axon regrowth and provide intact longitudinal intraluminal guidance (Muheremu and Ao 2015; Sabongi et al. 2015; Du et al. 2018). Whereas autografts are clearly advantageous in terms of biocompatibility, they also present major obstacles, such as limited availability of transplant material, inadequacy of the graft with the size of the lesion, graft donor-site morbidity, potential permanent loss of nerve function, need for a second surgical exposure, and possible occurrence of painful neuromas at the site of the injury. These limitations have given rise to the need for research and development of alternative treatments such as artificial (biological or synthetic) conduits or scaffolds, some of which very promising (Suematsu et al. 1988; Chiu and Strauch 1990; Crouzier et al. 2009; Deal et al. 2012; Sabongi et al. 2015; Hansen et al. 2016; Chato-Astrain et al. 2018; Han et al. 2019). To optimize nerve regeneration, those scaffolds must own chemical and physical properties that mimic their physiological environment (Evans et al. 1995; Chen et al. 2007; Barrette et al. 2008). Conduits are tubular structures designed to bridge nerve stumps over the gap, protect the injured area and prevent scarring, accumulate neurotrophic factors locally and mechanically guide regenerating axons (Jiang et al. 2010; Konofaos and Ver Halen 2013). Among those guides, vein tubes have been shown to be operative in bridging small nerve defects both in animals (Suematsu et al. 1998; Archibald et al. 1995) and humans (Chiu and Strauch 1990).

Human perinatal tissues have been used in regenerative medicine for over a century as allogeneic biomaterials, due to their advantageous properties including low immunogenicity and other immune privileges (Moore et al. 2017; Arutyunyan et al. 2018). Umbilical cord (UC) is a tube-like structure easily available although generally wasted, which encloses one vein and two arteries buried within a protective glycoprotein-rich extracellular matrix called the Wharton's jelly. In this study, we investigate the use of an inverted human UC artery (iHUA) as a tridimensional (3D) scaffold, offering a unique mechanical and chemical environment to damaged nerve ends, to improve nerve regeneration. The properties of this tissue-engineered biomaterial, combined with the straightforwardness of its handling and the simplification of logistical and surgical procedures, make its use attractive for the development of innovative regenerative therapies with better outcomes for patients suffering from PNI.

## Materials and methods

### Inverted human umbilical cord artery (iHUA) preparation

The experimental item tested in this study is a human umbilical cord artery (HUA) surrounded by a glycoprotein-rich Wharton’s jelly, which was inverted (inverted human UC artery: iHUA) in order to display the vessel wall on the outside, and the Wharton’s jelly on the inside. The requisite to inverse the structure comes from the fact that the conduit is, this way, more resistant to collapsing, furthermore offering a rich collagen matrix directly in contact with the regenerative axons within the nerve chamber (Wang et al. 1993; Ferrari et al. 1999; Crouzier et al. 2009). Concisely, human UCs were collected after the completion of a socio-clinical questionnaire of pregnant mothers and patients consent according to Directives 2006/17/EC and 2006/86/EC. In addition, qualification criteria were based on the absence of risks during pregnancy, a baby birth weight greater than 2.8 kg. As soon as the baby was born, blood sample was collected to validate a negative serology for human immunodeficiency virus (HIV), human T-lymphotropic virus (HTLV), hepatitis B virus (HBV), hepatitis C virus (HCV) and syphilis. UCs were sampled from maternity unit during childbirth and shipped at + 4 °C in 0.9% NaCl to the tissue Bank within 24 h of birth delivery, where they were carefully registered and the media checked for possible risks of contamination. After reception at the Tissue Bank (TBF), validation of clinical criteria of donation and registration of unique donation codes, UCs were then immersed in sterile water for a minimum of 2 h so that it can be trapped by proteoglycans. Afterwards, arteries surrounded by the protective Wharton’s jelly were mechanically isolated, cut into pieces, before the segment was turned inside-out (iHUA) by a technique inspired by sewing instrumentation (steel loop turner). iHUAs were kept frozen at − 25 °C, and a sterile polytetrafluoroethylene (PTFE) guide adapted to 1.75 mm diameter sections was inserted inside each tubular structure. At the time of preparation, iHUAs were soaked in purified water, then in 70% (v/v) ethanol for 1 h and hydrogen peroxide (30%—15 min then 3%—1 h) for virus inactivation (validated according to a > 5-log decrease in porcine parvovirus (PPV), pseudorabies virus (PrV), HIV and hepatovirus A (HAV)). Due to the fact that fetal tissue has a low immunogenicity reaction, no RNA extraction was included in the chemical process.

After final washes in buffer and in deionized water, iHUAs were lyophilized on the same sterile PTFE guides adapted to 1.75 mm diameter sections. The guides were then removed and iHUAs were cut into 2 cm long segments, packed in double pouches and the product was irradiated at 25 to 32 kGray. iHUAs were stored at room temperature prior to their subsequent use as 3D scaffold nerve chambers for the repair of peripheral nerve damage.

**Fig. 1:**
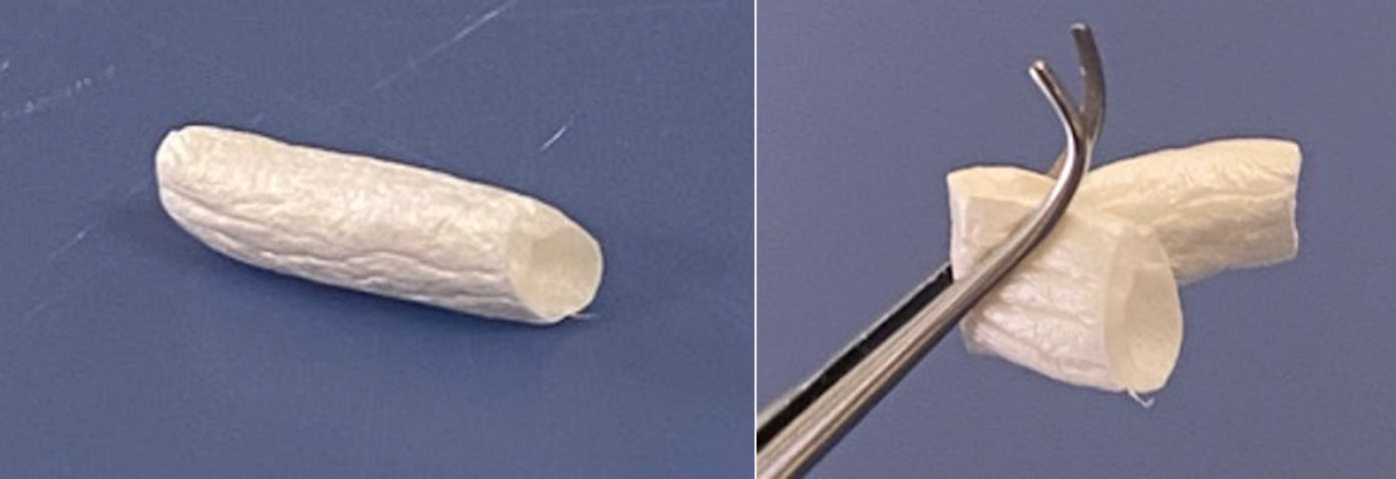
Macroscopical structure of the inverted vessel product (NerVFIX; Tissue Bank France, Mions, France)

**Fig. 2:**
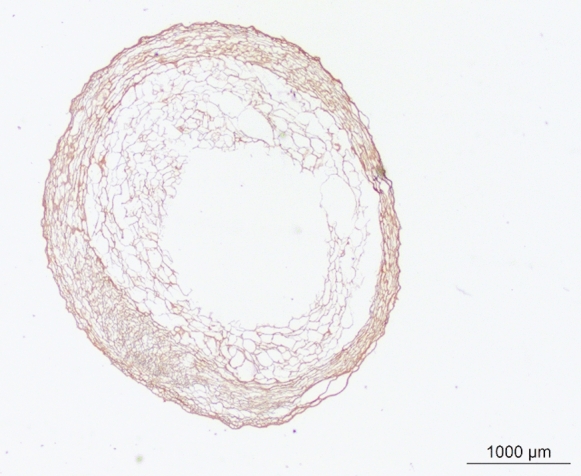
Collagen IV in the structure of the layer of the vascular wall identified with collagen IV antibody (20411—Purified freeze-dried antibody to human type IV collagen; Novotec, Bron, France). The vascular wall is external, the Wharton’s jelly is internal. The Wharton Jelly appears as a porous structure with reticular collagen

Macroscopically, the inverted vessel treated by this process is a flexible tubular structure (Fig. 1). The validation of the devitalization and of the structure was done histologically with persistence of dead cells. Umbilical vessel structure was preserved even after turning out with elastin, collagen IV and laminin in the basement membrane of the vascular wall. Similarly, Wharton’s Jelly structure was preserved with collagen I, elastin and a high level of proteoglycan and hyaluronic acid (Fig. 2). Culture of mesenchymental stem cells in contact of iHUA shown integration in the structure and multiplication. Testing following ISO 10993 standards validated the good biocompatibility.

### Animals

Fifteen (15) healthy male Sprague–Dawley rats over 12 weeks-old of approximately 200–250 g were obtained from Janvier Labs (CS 4105 Le Genest Saint Isle, F-53941 Saint-Berthevin Cedex, France) and kept under veterinary and technical supervision at Atlantera preclinical center, the animal facilities of Atlantic Bone Screen (Saint-Herblain, France). Animals were housed in plastic boxes of standard dimensions under controlled conditions of temperature and light cycles (20 ± 1 °C, 12/12 h light/dark), with ad libitum access to rat food and tap water. All experimental procedures were carried out by Atlantic Bone Screen in accordance with the current legislation (Decree N2013-118 of February 1st, 2013 on animals used in experimental purposes). The use of animals for scientific purposes in this study was approved by the French Minister of Higher Education, Research and Innovation (APAFiS-2163).

Animals were acclimatized for at least 7 days prior to the surgery. Surgery was performed under aseptic conditions on animals deeply anesthetized, and all efforts were made with local analgesia to minimize their pain and distress at all time. Rats were subsequently accommodated individually and their clinical status was daily monitored throughout the all experimental period until they were sacrificed.

### Study design

Animal studies and procedures complied with applicable international, national and institutional guidelines for the care and use of animals.

The study design is based on works previously described in the literature (Godinho et al. 2013; Carriel et al. 2014; Cheng et al. 2015; Li et al. 2016, 2017). All animals (n = 15) were subjected to general anesthesia before undergoing the same surgical procedure. Transection of the sciatic nerve (SN) was performed in their right leg to generate a gap of approximately 1 cm long, followed by the implantation of an iHUA segment used as a nerve chamber sutured on both sides of the resected nerve of the host. The extremities of nerve section were suture with 9.0 nylon epineural stitches. The needle was passed through the non-rehydrated 1.5 cm long iHUA. The iHUA was then slid on the sciatic nerve to cover the gap. Both extremities were epineurally sutured to the iHUA. Rats were randomly assigned to three (3) groups (n = 5/group) conditioning the date of their surgery and sacrifice. Five animals sacrificed at “EARLY time” (7 days/1 week), five at “INTERMEDIATE time” (21 days/3 weeks) and five at “LATE time” (56 days/8 weeks) after surgery.

Nerve regrowth after implantation of the iHUA insert was investigated over time by clinical examination and functional tests assessment of SN regeneration, combined with the evaluation of gastrocnemius muscle (GM) atrophy. Blind analysis of histology with grade of the local tolerance (assessment of local tissue reaction, LTR) and insert biodegradability (IBD), completed by thorough study of the quality of the sciatic nerve reconstruction (SNR) along the graft (% of assembly of newly formed axons, myelin sheath, Büngner bands organization and quantity of glial tissue formation) were performed. Likewise, histomorphometric analyses of SN regeneration at different locations of the nerve chamber, along with GMs transversal section explorations were implemented to complete this study.

### Procedures

Animals were subjected to general anesthesia according to Atlantic Bone Screen internal standard operation procedures and analgesic procedure was applied 30 min to 1 h before surgery.

Complete SN transection was performed according to a standard procedure described in the literature (Cheng et al. 2015). Anesthetized rats were placed in the prone position. The right hindlimb was shaved and carefully disinfected with an iodophor. A skin incision was made in the posterolateral right hindlimb, and the intermuscular septum was bluntly dissected between the gluteus maximus and the femoral biceps. Using a light microscope at 4 × magnification (Leica, 10446294), the SN was exposed and dissociated, and a 1.0 cm segment was transected completely with micro scissors.

Dried iHUA was previously trimmed to reach 1.5 cm in length, before being implanted, under the microscope, at the location of the nerve transection. Thus, the iHUAs hereby used as a nerve chamber, was stitched to the host opposite nerve stumps using a 9–0 noninvasive suture with 2 mm overlapping between the nerve section and the iHUA, and a final gap of 1.0 cm. The nerve chamber was then rehydrated with sterile saline solution (NaCl 0.9%). Afterwards, the SN was placed back between the two muscles and the skin wound was sutured with surgical staples.

#### Clinical follow-up

Besides daily monitoring of animal wellbeing, a thorough clinical follow up of the animals vital parameters, signs of seizure, motor activity and behavior, infection and/or sores at the operated area was performed three times a week during the over experimental period. Body weight was also recorded twice a week. Evaluation of the SN functionality consisted in (1) evaluating the sensitivity of the leg by monitoring the reflex index (withdrawal reaction) after a mild pinching stimulus applied to the skin of the right hind-paw; (2) scoring the motricity index by following the tonicity of the operated leg in the emptiness and while climbing. All parameters were graded from 0 to 2 by comparing the reaction of the right hindlimb with the responsiveness of the healthy left one, with 0 corresponding to no reaction and 2 corresponding to the same reaction as healthy hindlimb.

#### Sampling procedure

Animals were sacrificed at different time points following the surgery [“EARLY time” = Day 7 (n = 5), “INTERMEDIATE time” = Day 21 (n = 5) and “LATE time” = Day 56 (n = 5)], by cervical dislocation under anesthesia preceded by exsanguination. A rapid autopsy was performed on each animal.

The right operated SN and adjacent tissue of each rat was removed, along with the intact left SN of only one animal from each time point. Specimens were stored individually in 10% (v/v) formalin solution. GMs from both hindlimbs were collected from each rat on their corresponding date of sacrifice. Before being fixed, GMs mass ratio was assessed. Overall, 18 SNs (right, n = 15; left, n = 3 (1/time point)) and 30 GMs (right, n = 15; left, n = 15) were collected for further analysis.

#### Macroscopic evaluation at the nerve chamber implantation site

Macroscopic analysis was aimed to evaluate nerve continuity, uniformity, adherences or inflammation reactions at different times (early, intermediate and late) following the reconstructive surgery. LTR was monitored observing hematoma/bleeding, edema, fibrous reaction (adherence to the adjacent tissue), tissue necrosis and erythema/congestion formation at the location of the nerve chamber insertion after SN transection. The evaluation of those parameters was done by 2 observers and scored individually, according to the severity of the reaction, on a scale of intensity ranging from 0 (absence) to 3 (severe extent). The mean score of each parameter was calculated and the sum of the mean score of each parameter was presented as the total mean score of the LTR at its corresponding recuperation time after surgery.

#### Tissue processing for histological analysis

Collected samples were fixed in 10% formalin neutral buffered, for 48 h (SN) to 1 week (GM). All fixed tissues were oriented and routinely processed for paraffin embedding, sliced into 4 µm thick sections with a rotary or sliding microtome. Slices were further treated to attach to a glass slide, and thoroughly dried overnight before histological processing. For SNs, two (2) longitudinal sections were made at the nerve chamber insertion site (thus displaying the transected area and the nerve stumps on both sides) approximately 150 µm apart. For GMs assessment, transversal sections at the center of the muscle were prepared.

General histology was assessed by conventional hematoxylin–eosin (HE) staining. In addition, MCOLL histochemical method (Carriel et al. 2011), combining Luxol Fast Blue (LFB) and PicroSirius Red staining, was used to evaluate remyelination and collagen fibers reorganization processes. The myelin sheaths are stained in blue, the collagen fibers in dark red, the nuclei of the different cell populations in violet, and the tissue background in pink. The presence of Schwann cells and newly formed axonal sprouts were evaluated by indirect immunohistochemistry (IHC) for S-100 (Diagomics, Rabbit anti-S-100 (Z031129-2)) and neurofilament (NF; Diagomics, Mouse anti-NF (N2912)) proteins, respectively, as previously described (Carriel et al. 2014).

The Schwann cells are stained in brown with anti-S-100 antibody. The NF appears as a marker of mature axons and is expressed strongly in the axoplasm in brown.

Details of the parameters taken into consideration for the histopathological analysis of SN reconstruction are presented in Table 1. The iHUA graft tolerance was evaluated by following the LTR. The LTR was calculated as the sum of the inflammation, the edema/congestion, the necrosis and the fibroplasia. Each of the 4 parameters constituting the LTR was scored on a scale from 0 (absence) to 5 (severe extend). The IBD was followed by monitoring the quantity of iHUA remaining residues over time compared to the original size of the insert, and the amount of cell colonization surrounding the area.

**Table 1:** Histopathological analysis parameters and their rating. Histopathological observations were carried out on HE, MCOLL, and IHC staining (NF and S-100 markers) of SNs longitudinal sections. Each parameter was quantified individually in a scale of intensity ranging from 0 to 5. Unless specified (**♣** n = 1), those observations were made on 2 different longitudinal sections of the SN separated from each other by approximately 150 µm within the nerve chamber. SNR was assessed by studying: the density, orientation, thickness and length of the newly formed axons; the intensity, thickness and length of the myelin formation covering these axons; and the density of the Schwann cells proliferating on site, and their organization in glial bands

Newly formed axons	Density of newly formed axons	0	No axons	3	Moderate
		1	Minimal	4	Marked
		2	Mild	5	High
	Orientation (axons in longitudinal orientation)	0	No axons	3	Moderate proportion of axons
		1	Very poor orientation of axons	4	Majority of axons
		2	Minority of axons	5	Perfect orientation of axons
	Thickness	0	No axons	2	Normal thickness
		1	Thin regrowing axons		
	Length (*axonal regrowth into the void*)	0	No axons	3	< 50% of the void;
		1	Very short (proximal edge)	4	> 50% of the void;
		2	Short (focal at proximal edge)	5	Entire void length
Myelination	Intensity ^♣^	0	No myelin sheaths	3	Moderate
		1	Minimal	4	Marked
		2	Mild	5	High
	Thickness	0	No myelin sheaths	2	Normal myelin sheaths
		1	Thin myelin sheaths		
	Length ^♣^(*into the void*)	0	No myelin sheaths	3	< 50% (up to half of the void)
		1	Minimal (limited to edge)	4	> 50% of the void
		2	Short (proximal edge)	5	Entire void length
Schwann cells	Density ^♣^(*proliferation into the void*)	0	No cells	3	Moderate density (within void)
		1	Rare cells (proximal edge)	4	High density (within void)
		2	Few cells (proximal edge)	5	Very high density filling the void
	Glial bands of Büngner formation ^♣^	0	No cells	3	Moderate quantity
		1	Isolated cells	4	Numerous/long glial bands
		2	Few/short glial bands	5	Glial bands filling the void

#### Histomorphometric assessment

In order to validate the semi-quantitative evaluations of the different parameters assessed from the histological analysis, a histomorphometric assessment was performed from the SN and GM samples.

For SNs, the extent of the surface occupied over time by mature axons and glial Büngner bands (Schwann cells) has been calculated by quantifying NF and S-100 proteins staining, respectively, at proximal, central and distal areas of SN stumps. Image capture and analysis were performed with ImageJ software (https://imagej.net. Accessed 19 August 2021). In order to separate the HE coloration from the immunostaining, HE DAB-plug-in was applied to separate the color-specific canals from the original pictures. Images thus generated by color subtraction were used to assess de IHC specific signal. Thresholds were adapted to each IHC signal (0–165 for NF and 0–175 for S-100 staining respectively). Results expressed in pixels were further converted in mm square (mm^2^) of stained surface. Area quantification at a given time point after surgery is presented as the mean surface (mm^2^) ± standard deviation (SD) from two (2) longitudinal sections of the nerve, at proximal, central and distal parts of the defect as well as surrounding (around) and within (into) the nerve chamber.

### Results presentation and statistical analysis

All semi-quantitative data obtained from macroscopic observations (clinical and functional assessments, SN and GM histomorphometric assessments) are expressed as the mean score ± standard deviation (mean ± SD) calculated on their respective scales at their specific recuperation time after the reconstructive surgery. For the parameters of interest these individual observations are referring to, results are expressed as mean total score (sum of individual mean scores) ± SD.

More specifically, mean figures from the histopathological analysis are calculated from the scoring of individual observations from one (1) or (2) longitudinal sections of the SN obtained from animals (n = 5) sacrificed at different time after the surgery (“EARLY”, “INTERMEDIATE” and “LATE”). Mean total score ± SD of each parameter of interest is presented as the sum of individual mean scores these are referring to.

## Results

### General condition of rats after surgery

After transection of the SN of right hindlimb, paralysis of the leg was visible. During the clinical follow-up of the animals, we observed self-mutilation behavior in 60% of the rats starting from day 10. Therefore, animals showing this conduct (n = 9) were treated with a daily dose of local analgesia (2% Lidocaine / 0.5% Bupivacaine (v/v) solution) administrated by subcutaneous injection in the right femoral area, until sacrificed. However, due to the severity of the self-injuries, 3 animals were sacrificed ahead of time: 2 animals from the intermediate time were sacrificed at day 11 and day 12 and were transferred in the group of early time, one animal for the late stage was sacrificed at day 48. In addition, operation procedure was modified for second and third scheduled surgery groups to decrease the risk of self-mutilation behavior. For those 2 groups, a drop of 2% Lidocaine / 0.5% Bupivacaine (v/v) solution was applied for 5 min on the exposed SN before being extensively washed with a saline solution. In order to avoid nerve compression, the SN transection was then performed with a lancet instead of micro scissors.

According to Challa (2015), SN transection simulates the clinical symptoms of “phantom limb”, producing neuropathic pain in the absence of any sensory input. This conduct confirms the effective transection of the right SN. No other clinical signs were observed during the course of the experimentation.

### Assessment of the functional recovery of the sciatic nerve

Functional recovery of the SN over time was assessed by performing sensitivity and motricity tests from first week (n = 15) to 8th week (n = 5) following the grafting of the iHUA segment. One rat from the “EARLY” group shown tonus into the emptiness and by climbing as well as reflex from day 1 and was suspected to have undergone a partial SN transection. First signs of reflex retraction subsequent to pinching were observed at day 5 in 47% of the animals. The recovery of the reflex and the tonus of the leg while climbing increased over time and reached a plateau in the third week (reflex: mean score = 1.2 ± 0.4; tonus: mean score = 1.4 ± 0.5) before increasing again to reach a mean score of 1.7 ± 0.6 for the reflex and a mean score = 2.0 ± 0.0 for the tonus at 8 weeks. Therefore, the reflex recovery of the right hindlimb, compared to the left one, was not total at that stage (85%). First signs of tonus of the leg in the emptiness appeared much later (day 24) and were of weak intensity (10%). A maximum recovery of 25% was observed at 7 weeks (mean score = 0.5 ± 0.6). Overall, the global score of those functional tests is in favor of general SN regeneration over time, starting from day 5 (20% recovery). However, the recovery is not complete; mean total score of 3.7 ± 0.6 observed at the end of the study thus representing a recovery of 62%; mainly due to a poor tonus in the emptiness recovery.

### Macroscopic observations at the site of the intervention

In order to study the LTR of the SN to the iHUA graft over time, animals were sacrificed at different times (“EARLY”: 7 (n = 3), 11 (n = 1) and 12 (n = 1) days; “INTERMEDIATE”: 21 (n = 5) days; and “LATE”: 48 (n = 1) and 56 (n = 4) days) following the surgery. The autopsies did not highlight any organ lesion. No external signs of local infection of the wound were detected either.

Macroscopic observations of the SN at the site of the intervention were performed, recording local signs of hematoma/bleeding, edema, fibrous reaction, tissue necrosis and erythema/congestion. No hematoma or tissue necrosis was detected during the entire study. A fibrous reaction of moderate intensity, associated with mild marks of erythema and minor symptoms of edema, was observed at early stage. This fibrous reaction was associated to the slight adherence of the adjacent connective tissue to the nerve chamber, which disappeared over time. No signs of inflammation or chamber degradation were observed at intermediate and late stages.

### Histopathology

#### Local tolerance and insert biodegradability

LTR was assessed by monitoring inflammation, edema/congestion, necrosis and fibroplasia at early, intermediate and late stages. An inflammatory response was observed in all rats although the marks decreased over time and mild symptoms persisted at the end of the study. Infiltrates were mainly distributed at the periphery of the nerve chamber and around the nerve stumps at early time and got smaller and diffused later on. Local edema of minimal intensity, due to the intervention itself, was present after first week, but disappeared completely over time. Trivial signs of fibroplasia were still present at late stage, confirming our preliminary macroscopic *post-mortem* observations. No necrosis was reported. Overall, the LTR was satisfactory from the early stages following the reconstructive surgery, reflecting a good local tolerance of the iHUA insert maintained over time (from 70% at early stage to 80% at late stage).

Likewise, the IBD was monitored over time by scoring the quantity of remaining iHUA residues at the site of its graft, and the amount of cell colonization in contact with those residues. At early stage, the nerve chamber remained almost intact, although its size decreased over time, especially between the early and intermediate stages when two-thirds disappeared (34% remaining). At late stage, the nerve chamber is highly degraded in all rats, with only 20% of residues still detected.

#### Nerve reconstruction

Cell colonization increases over time. Indeed, a central hypocellular structure (thick acellular fibrous layers) covered on the outside by cell colonization siting in-between proximal and distal regenerative cones in formation, was observed at early stage. This assembly was progressively substituted by central nerve regeneration, with rare and disseminated inflammatory foci.

**Fig. 3:**
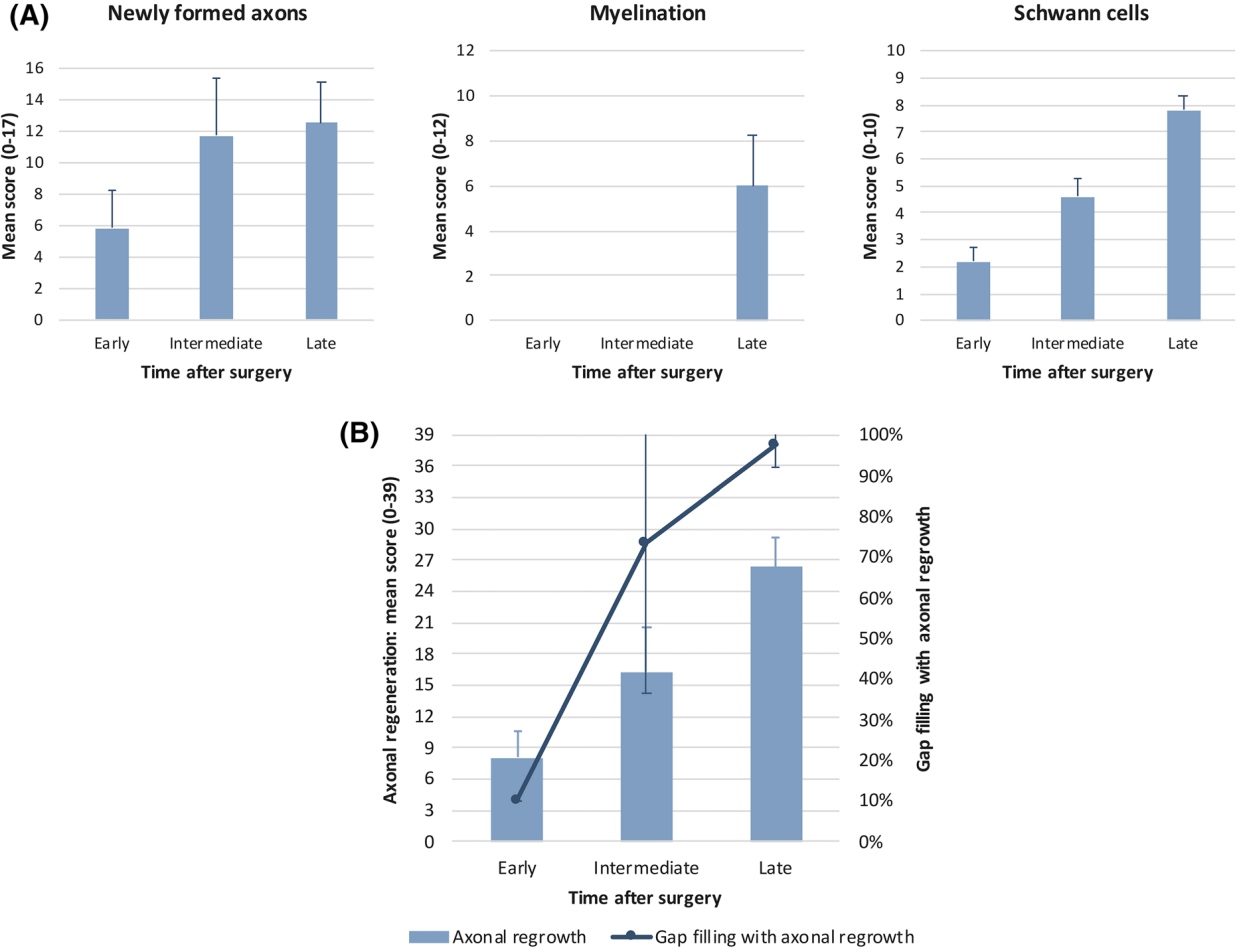
Monitoring of a post-traumatic SNR after implantation of the iHUA chamber. **a** Evaluation of individual parameters of SNR over time. Results are expressed as mean score ± SD of the newly formed axons, myelination and Schwann cells; **b** mean score of axonal regeneration ± SD was calculated at different times after suturing the iHUA nerve chamber to the transected sciatic nerve and is expressed as the sum of the individual total mean scores of newly formed axons, myelination and Schwann cells formation (score 0–39). The % of gap filling was estimated as the percentage of nervous reconstruction length in the void. The estimations were made on 2 different longitudinal sections of the SN separated from each other by approximately 150 µm within the nerve chamber

SNR in the presence of the iHUA insert was assessed over time by histological analysis, following newly formed axons physical characteristics and their myelination, together with the recolonization of the damaged area by Schwann cells and their progressive organization in regeneration tracks (Büngner bands) essential for guiding the regrowing axon during the extended period of nerve repair. Semi-quantitative evaluations of those factors are presented in Fig. 3a. Overall, SNR progressively increased over time as illustrated by the mean axonal regeneration score presented in Fig. 3b. The axon gap filling occupancy connecting both stumps, reaches 98% after 8 weeks following the surgery, although the nerve is still immature with a low amount of myelination.

**Fig. 4:**
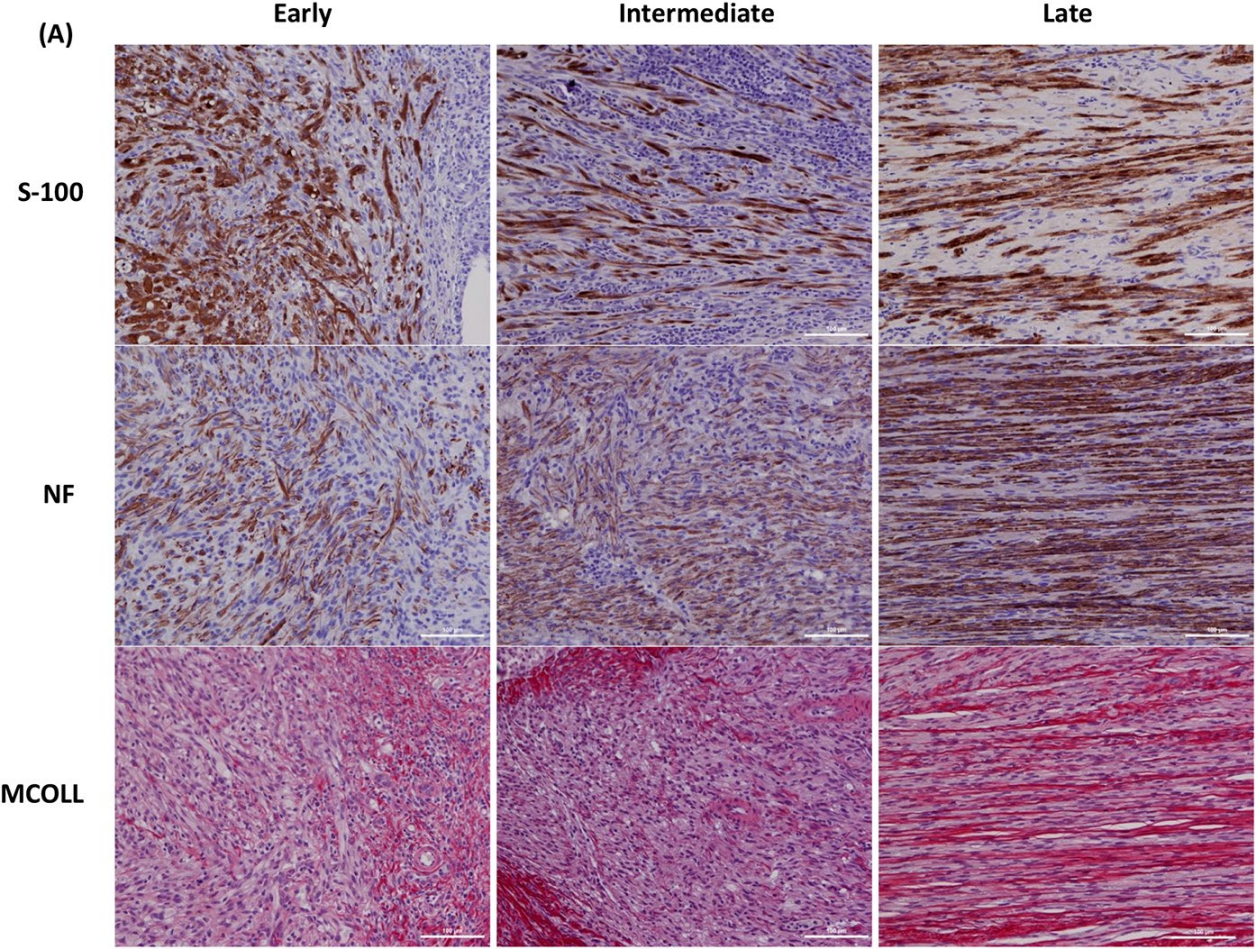
Overview of SNR over time. Longitudinal sections proximal extremity of the SN at different times (early, intermediate and late) after the graft of iHUA insert was stained with S-100 (Schwann Cells), NF (axonal sprouting) and MCOLL (myelin) (scale bars: 100 µm)

Longitudinal sections of the SN illustrating the regeneration process over time are presented in Fig. 4. At day 7, tiny newly formed axons expand in random direction from regeneration cones formed at the proximal extremity of the SN. Most grow along the graft, but not inside the porous structure of the nerve chamber remaining hypocellular at that stage. Since day 21, axon regrowth filled the grafted area, the nerve chamber being almost fully integrated with only few remaining residues. They gradually elongate and form a dense regular network oriented parallel to the nerve chamber, which eventually fill the entire width of the gap as observed at day 56. Mild proliferation of Schwann cells was also registered in early days, with a distribution pattern similar to that of the newly formed axons. Schwann cells density increased over time, forming thicker and longer glial bands through the gap. Myelination of low intensity, limited to proximal edges or focally spread along the newly formed axons, was only seen at late stage of the regeneration process. Moreover, typical feature of Wallerian degeneration was observed since early stage at the distal extremity of the injured SN. At late time, some newly formed axons are identified in the area, indicating that anastomosis between both SN extremities is effective.

### Histomorphometry

#### Sciatic nerve recovery

**Fig. 5:**
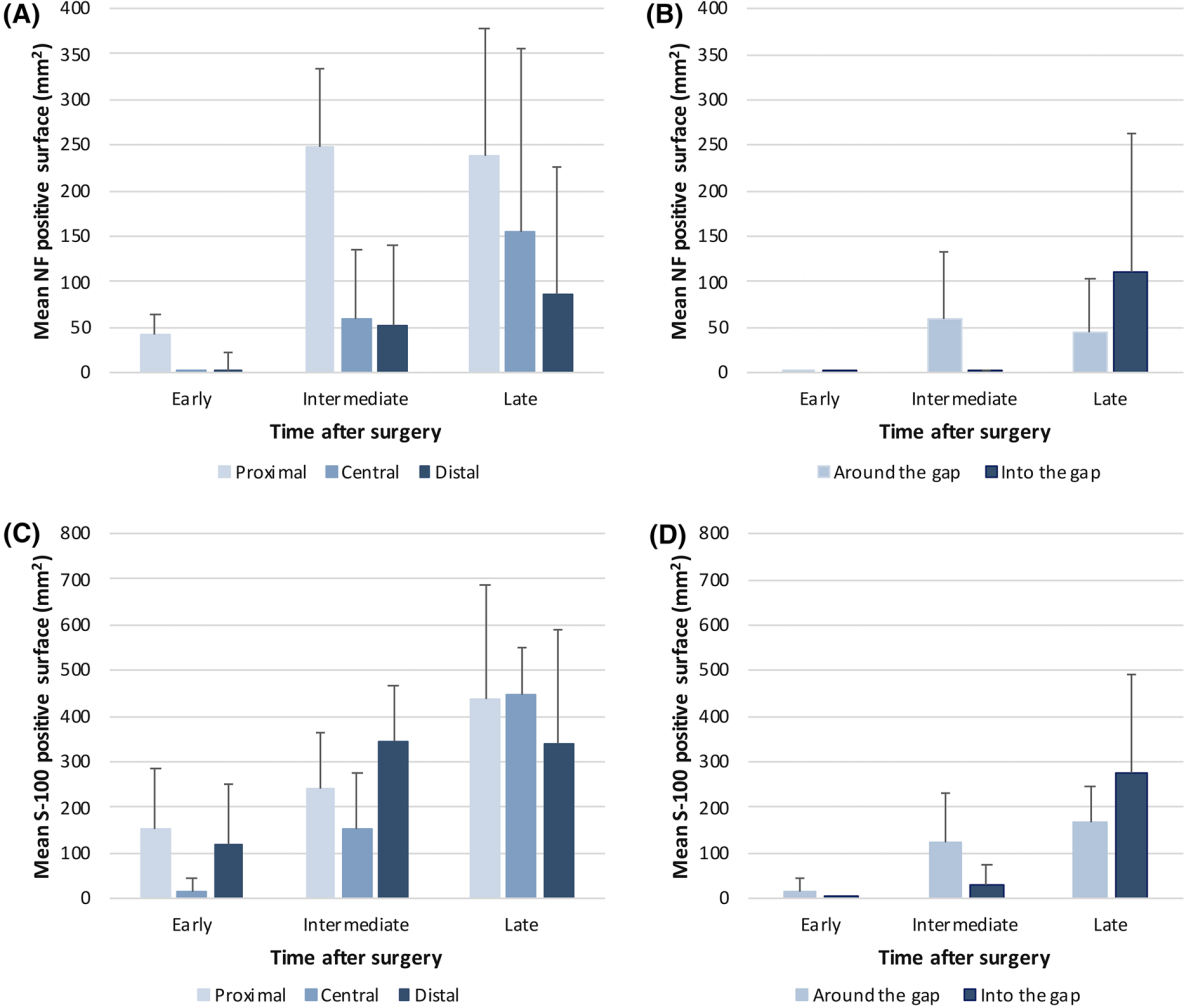
Histomorphometric assessments. Mean surface coverage (mm^2^) ± SD of NF (newly formed axons marker) over time at **a** proximal, central and distal parts of the lesion and **b** around and inside the iHUA insert; Mean surface coverage (mm^2^) ± SD of S-100 (Schwann cells marker) over time at **c** proximal, central and distal parts of the lesion and **d** around and inside the iHUA insert

In addition to histopathological analyses, measurements were made to quantify the area occupied by mature axons expressing NF marker and Schwann cells (S-100 marker), at the proximal, central and distal parts of the lesion (Fig. 5a, c). Besides, the spatial localization of their respective distributions was investigated in more details around and inside the iHUA insert (Fig. 5b, d). As expected, the newly formed axons started to expand from the proximal stump of the transected SN. The NF-stained surface within the proximal area increased over time to reach a plateau at day 21, and progressively expanded in the central and distal zones of the lesion. In the center, axons were mainly present externally and progressively invaded the nerve chamber. These results are consistent with the histopathologic analyses showing a progressive axonal regrowth from proximal towards distal stumps. Histomorphometric analysis of the surface occupied by Schwann cells (S-100 staining) showed that cells were present at both extremities at early stage, and that some were already invading the center of the injured SN. The presence of Schwann cells at the distal stumps and their active expansion over time is consistent with their involvement in the Wallerian degeneration observed during the process of SNR. In the central zone, those cells were present since early stage on the outside part of the nerve chamber, and progressively invaded it. Those features are consistent with histopathologic observations, where the density of Schwann cells was seen to increase to form thicker and longer glial bands (Büngner bands) within the entire gap, following the distribution pattern of the growing axons of which Schwann cells orchestrate the myelination.

## Discussion

Posttraumatic nerve repair is one of the major challenges in regenerative medicine and microsurgery. Despite the recent progresses in the field of tissue engineering, functional recovery after severe nerve lesions is generally partial and unsatisfactory. Autologous nerve graft is still the best method to treat peripheral nerve lesions (Tezcan 2017; Ferrante 2018), although it has several drawbacks and does not allow complete functional recovery. Extensive research has suggested that complete recovery of nerve functionality could ideally be achieved by guiding axon regeneration toward its original tissue target, using intraluminal nerve channels (Muheremu and Ao 2015; Sabongi et al. 2015; Du et al. 2018). In the last decades, a wide variety of artificial nerve guides of natural or synthetic origins have been engineered to be used as bioactive fillers, porous and non-porous, biodegradables or not, combining their use with the addition of essential neurotrophic growth factors or supportive cells, some of which approved for clinical applications (Chiu and Strauch 1990; Schmidt and Leach 2003; Crouzier et al. 2009; Deal et al. 2012; Sabongi et al. 2015; Tezcan 2017; Chato-Astrain et al. 2018; Han et al. 2019). The current trend is the development of biomimetic nerve guides capable of providing topological, chemotactic and haptotactic signaling to the inner cells involved in the nerve repair process. In order to enhance peripheral nerve regeneration outcomes in the presence of artificial conduits, a better understanding of the underlying mechanisms of repair is required. In this study, we investigate the use of an inverted human umbilical cord artery (iHUA) as a 3D scaffold nerve chamber for improving peripheral nerve regeneration after complete SN transection in rat.

The iHUA insert was well tolerated at the implantation site and was, on average, degraded at 80% within 8 weeks. Chamber residues did not induce any significant necrosis, and only a low degree of inflammation, combined with some edema and congestion, was observed at early stage but disappeared afterwards. Fibrosis of moderate intensity was initially detected and therefore considered as a beneficial scaffold for nerve regrowth and integration of the implant. A progressive nerve chamber integration to the tissues (cell colonization and iHUA insert degradation), concomitant to the nerve reconstruction process, was observed over 8 weeks. A progressive bridging of the transected stumps of the SN has been observed over time. On day 7, tiny newly formed axons expand in random direction from the proximal regeneration cones, embedding the outside of the chamber. After 3 weeks, axons regrowth filled the inner part of grafted area, and gradually elongate towards the distal stump, forming a dense network of regular arrangement, oriented parallel to the nerve chamber, close to the standard aspect of a nerve fascicle. After 8 weeks, the axon gap filling occupancy connecting both stumps was almost complete, demonstrating that the anastomosis is effective.

Schwann cells play a pivotal role in the selective promotion of motor and sensory axon regeneration, forming Büngner bands within nerves, hence providing a guidance substrate for the re-growth of axons of which they orchestrate the remyelination (Napoli et al. 2012; Jessen and Mirsky 2016). They are also involved in the secretion of neurotrophic growth factors and cytokines, establishing a permissive local microenvironment for nerve regeneration (Chen et al. 2005; Webber and Zochodne 2010; Wang et al. 2013; Jessen and Mirsky 2016). From day 7, they were seen proliferating on both extremities of the wounded SN, and on the outer part of the nerve chamber which they also started to invade. Their active expansion at the distal stump, together with the typical feature of Wallerian degeneration observed from early stage and persisting for at least 2 weeks, are compatible with their role clearing debris through phagocytosis and recruiting macrophages, and their involvement in nerve stump breakdown to give way to newly regenerating axons (Menorca et al. 2013; Caillaud et al. 2019). Over time, the density of Schwann cells increased, and they gradually migrated to the center of the chamber where they got aligned to form thicker and longer glial bands covering the entire width, overlapping with that of growing axons. After 8 weeks, their distribution pattern was similar to the one observed in the intact collateral SN. However, a low yield of myelination, limited to the proximal edges or focally spread along the newly forms axons, was detected only late in 4/5 rats. These observations are in concordance with the data described in the literature, which stipulate that the myelination is generally incomplete at 8 and 12 weeks after peripheral nerve transection in rats (Suematsu et al. 1998). At that time, regenerating axons supported by Büngner bands were still immature but the SN reconstruction was still in progress, with minimal signs of local fibrosis and inflammation.

Although axonal regeneration and their remyelination occur naturally in the peripheral nervous system, axons often display thinner myelin sheaths and a reduced internodal length, leading to slower nerve conduction affecting sensitivity and motricity (Sherman and Brophy 2005). Indeed, the first signs of leg climbing motricity recovery were observed at day 3 in 27% of the rats, with a complete recovery after 8 weeks. Moreover, reflex retraction subsequent to a pinching stimulus was detected in 47% of the animals at 5 days and the sensitivity recovery reached 80% at 2 weeks. However, weak signals of motricity monitoring the tonus of the leg in the emptiness were detected much later (day 24). Overall, the recovery of SN function was partial after 8 weeks, reaching 62% of the responses recorded for the opposite healthy hind-paw. These results matched with our assessment of the neighboring GM atrophy over time. Indeed, it is well established that denervated muscles suffer from progressive degeneration (Evans et al. 1995; Navarro 2016). We observed a rapid and severe reduction of 29% at one week and 65% at three weeks of the GM weight and 23% at week one and 65% at three weeks of the GM volume, combined with a decrease in the diameter of the muscle fibers of 42% of their original size during the first 3 weeks following the intervention. From this time, GM deterioration slow down; reduction of 77% and 74% of the GM weight and volume, respectively, and of 55% of the fibers diameter at 8 weeks; suggesting the launch of muscle innervation recovery (preparation of the 2 stumps of the injured SN, directed anastomosis with elongation of axons regrowth from the proximal to the distal end, and beginning of their progressive remyelination) confirmed by our histological observations.

Overall, the iHUA can be used as a 3D scaffold to help peripheral nerve regeneration, preventing exuberant axonal sprouting at proximal ends and local fibrotic reactions. The insert is well tolerated and almost completely degraded after 8 weeks. From the beginning, it acts as an integrated nerve chamber leaving room to migrating cells proliferating and gradually colonizing its interior, favoring an oriented anastomosis by guiding regrowing axons in a dense and aligned network progressively covered with a tick myelin sheath. At the end of our study, the SNR is incomplete (axons are still immature) and the GM atrophy still present; however, signs of functional recovery of the leg were detected.

## Data Availability

The datasets used and/or analyzed during the current study are available from the corresponding author on reasonable request.
